# Acupuncture for infantile colic: A blinding-validated, randomized controlled multicentre trial in general practice

**DOI:** 10.3109/02813432.2013.862915

**Published:** 2013-12

**Authors:** Holgeir Skjeie, Trygve Skonnord, Arne Fetveit, Mette Brekke

**Affiliations:** Department of General Practice, Institute of Health and Society, University of Oslo, Norway

**Keywords:** Acupuncture, acupuncture point, general practice, infantile colic, Norway, randomized controlled trial

## Abstract

**Objective:**

Infantile colic is a painful condition in the first months of infancy. Acupuncture is used in Scandinavia as a treatment for infantile colic. A randomized controlled trial was carried out with the aim of testing the hypothesis that acupuncture treatment has a clinically relevant effect for this condition.

**Design:**

A prospective, blinding-validated, randomized controlled multicentre trial in general practice. Research assistants and parents were blinded.

**Setting:**

13 GPs’ offices in Southern Norway.

**Intervention:**

Three days of bilateral needling of the acupuncture point ST36, with no treatment as control.

**Subjects:**

113 patients were recruited; 23 patients were excluded, and 90 randomized; 79 diaries and 84 interviews were analysed.

**Main outcome measures:**

Difference in changes in crying time during the trial period between the intervention and control group.

**Results:**

The blinding validation questions showed a random distribution with p = 0.41 and 0.60, indicating true blinding. We found no statistically significant difference in crying time reduction between acupuncture and control group at any of the measured intervals, nor in the main analysis of differences in changes over time (p = 0.26). There was a tendency in favour of the acupuncture group, with a non-significant total baseline-corrected mean of 13 minutes (95% CI –24 to + 51) difference in crying time between the groups. This was not considered clinically relevant, according to protocol.

**Conclusion:**

This trial of acupuncture treatment for infantile colic showed no statistically significant or clinically relevant effect. With the current evidence, the authors suggest that acupuncture for infantile colic should be restricted to clinical trials.

- Acupuncture is used in Scandinavia as a treatment for infantile colic. This blinding-validated randomized controlled trial carried out in general practice found no statistically significant difference in duration of daily crying between a group subjected to acupuncture and a control group (p = 0.26).
- A non-significant 13-minute trend in favour of acupuncture was considered not to be clinically relevant.

## Introduction

Infantile colic is a painful and incompletely understood condition in the first months of infancy. The majority of studies of infantile colic have used the definition by Wessel et al: “Paroxystic uncontrollable crying and fussing in an otherwise healthy infant under three months of age, with more than three hours of crying per day in more than three days for more than three weeks.”[1]

Although infantile colic is a self-limiting condition, it is a severe strain on both the child and parents.[2] The aetiology is considered multifactorial. Possible mechanisms include physiological factors like painful intestinal contractions and altered gut motility, immaturity of gut function, lactose intolerance, food hypersensitivity, altered intestinal flora and gas, and psychological factors like inadequate mother–child interaction, anxiety, and infant temperament [3,4].

There is no consensus on treatment strategies for the condition, which include common strategies like hypoallergenic diet, soy formula, reduced stimulation, sucrose, and herbal tea [2,5]. Chiropractic manipulation has not shown effects in controlled studies.[6] Recently, administration of drops of specific Lactobacillus strains has shown promising results [7,8].

Acupuncture is a frequently used alternative treatment modality in Scandinavia [9] and is also used for infantile colic [10]. There is a well-founded concern for the ethics and the evidence concerning alternative treatment of paediatric conditions [11,12].

Acupuncture is an original Chinese treatment method using thin steel needles penetrating through the skin and into connective tissue and muscle fibres. The neurophysiologic basis for the observed effects, especially the pain-inhibiting effects, is relatively well understood.[13] Acupuncture is a safe procedure when used by trained practitioners, and the risk of serious adverse effects is low [14], in children also [15].

Two controlled trials of children with infantile colic treated with acupuncture have been published [16,17]. Both studies concluded that acupuncture significantly reduced crying and pain-related behaviour without noticeable adverse effects. Effect sizes were small, and there was no blinding validation.

General practitioners educated within the programmes of the Norwegian Society of Medical Acupuncture use a standardized bilateral needling of the point ST36 when treating infant colic. ST36 is located in the proximal part of anterior tibial muscle and is the acupuncture point considered most important for ailments of the gastro-intestinal apparatus in traditional Chinese medicine (TCM) [18,19]. A postulated neurophysiologic mechanism explains a beneficial effect on gut dysmotility by way of the parasympathetic vagal reflexes, as well as a centrally opioid-mediated pain inhibitory pathway [20].

We carried out a randomized controlled trial with the aim to test the hypothesis that such acupuncture treatment has an effect above no-treatment control in infantile colic. The main study was preceded by a pilot study [21].

## Material and methods

### Trial design, participants, and interventions

The study was a prospective, blinding-validated, multicentre, randomized controlled trial involving 13 GPs’ offices in Southern Norway. The trial was registered with Clinical Trial Registry Identifier NCT00907621.

The data collection was approved by the Norwegian Social Science Data Services (reference 21490/2/JE). Ethical approval was given by the Regional Ethics Committee of South-Eastern Norway (reference S-08732b 2008/17889s) and the trial was carried out in accordance with the Helsinki Declaration. The parents of the infants gave informed consent. The inclusion period was from September 2009 to December 2012. The participating doctors were all GP specialists with a minimum of 300 hours of acupuncture education and five years of practising acupuncture.

The patients fulfilled Wessel's criteria and were born at full term. They were randomized to active treatment or to no-treatment control. The assistant instructed the parents on how to fill in the crying registration form, and the patient was given appointments with the same GP three, four, and five days after inclusion.

The GP was alone in the treatment room with the infant during the intervention. The GP made a mark, 3 mm in diameter, at the point ST36 bilaterally on all children, to hide the insertion mark. In the intervention group, an ethylene-oxidised sterile Seirin acupuncture-needle (0.20 × 15 mm) was inserted at the acupuncture point ST36. The point was needled bilaterally to approximately 12 mm depth. The two needles were left inserted without manipulation for 30 seconds. The needles were then withdrawn and a waterproof circular adhesive dressing, to further hide the insertion area, was applied. An identical procedure, except for the needle insertions, was performed on each infant in the no-treatment control group. The same procedure was performed on days 4 and 5.

### Outcomes

The primary outcome was difference in changes in crying time in the registration period. Clinically relevant effect was defined as one-hour difference according to the pre-trial protocol. Secondary outcomes were differences in fulfilling Wessel's infantile colic criteria, the parents’ assessment of the child's condition, and adverse effects.

### Sample size, randomization, and blinding

We anticipated a standardized difference of 0.5 and a clinically relevant difference of one hour. With p < 0.05 and 80% power, we needed to include 120 infants. Randomization was done manually by two persons not otherwise involved in the study. Sealed, opaque, numbered envelopes were used. Randomization was closed until the start of the first intervention. The parents and assistants were blinded to the allocation. Blinding validation of the parents was done by two blinding validation questions, one immediately after the first intervention, the second at week four.

### Statistical methods

We used the software programs SPSS 19 and 20 for the statistical analyses. We used linear mixed models statistics for the analyses of the main outcome variable, and chi-square and Fisher's exact tests for secondary outcomes of categorical data, and blinding validation analyses.

## Results

### Participant flow and baseline data

**Figure 1.**
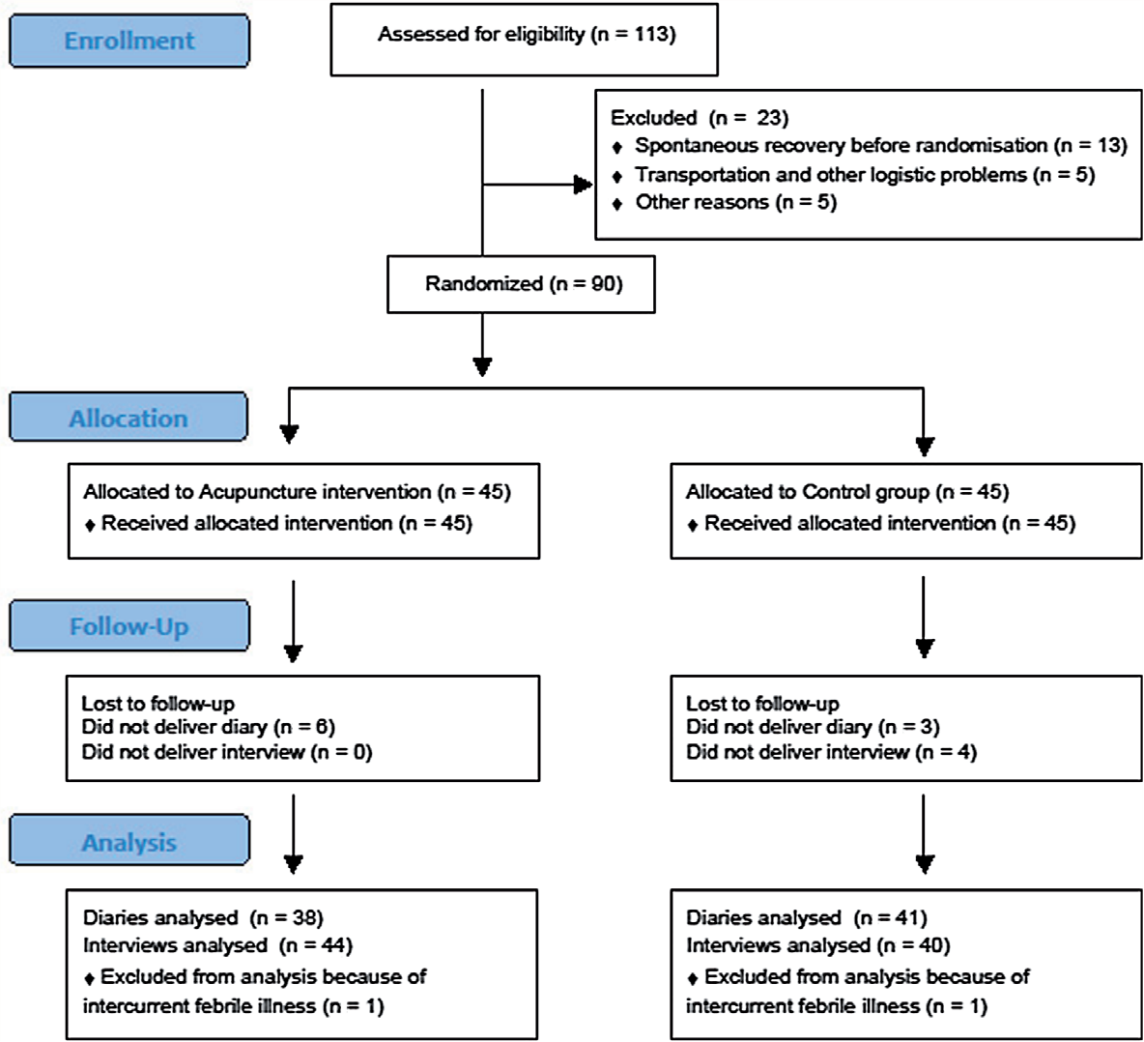
Trial flow chart: The ST36 infantile colic acupuncture trial.

A total of 113 patients were recruited; 23 patients were excluded, and 90 randomized; 79 diaries and 84 interviews were analysed. A flow-chart diagram is shown in Figure 1. For baseline data, see Table I.

**Table I.** Baseline characteristics: The ST36 infantile colic acupuncture trial.

	Acupuncture group (n = 44)	No treatment-control group (n = 40)
Sex-number of male/female	22/22	20/20
Mean age in weeks at inclusion (range)	6 (3–13)	6(3–9)
Mean gestation age in weeks at birth (range)	40 (36–42)	39 (36–42)
Mean birth weight in grams (range)	3590 (2580–4600)	3544 (2375–4410)
Feeding		
Breast	28	23
Supplement	9	5
Both breast and supplement	7	12
Pre-study interventions		
Chiropractic	6	5
Milk free diet in mother	4	2
Other (dimethichoner, herbal tea, malt extract, sugar water, massage)	9	*7*
Several different treatments tried	15	17
No pre-study treatments	10	9
Crying time	(n = 38)*	(n = 41)*
Baseline crying time in minutes (range)	220 (80–394)	212 (0–353)
Number of children with crying time ≥ 4 hours	12	17
Number of children with crying time < 4 hours	26	24

*The number of patients differ because of difference in numbers of interviews and reported crying diaries.

### Blinding validation

The primary blinding validation question “Do you think your child has received acupuncture or not?” was answered in 83 cases and showed a random distribution. In the acupuncture group 22 of the parents believed the child had acupuncture and 22 believed the child was in the control group. In the control group the numbers were 16 and 23 (p = 0.41). The second blinding validation question, “Have you noticed any needle insertion marks?”, was answered in 38 cases: 17 in the acupuncture group and 17 in the control group answered “No”. Three in the acupuncture group and one in the control group answered “yes” (p = 0.60). We thus consider the blinding as valid.

### Changes in crying time

**Figure 2.**
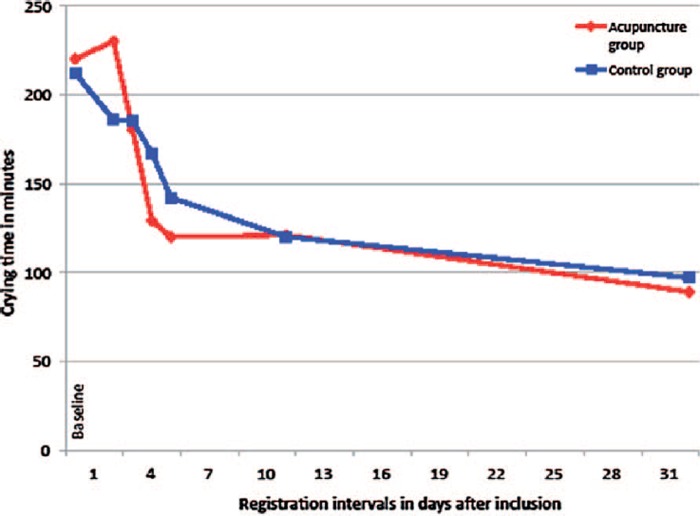
Plot diagram of changes in crying time: The ST36 infantile colic acupuncture trial.

The primary end point of the trial was the difference in crying time changes between the acupuncture group and the control group. Linear mixed model main analyses of interaction time versus group for primary end point gave p = 0.26. There was no statistically significant baseline-corrected difference for the whole period, (Table II) or at any measured time period from baseline to the last measure after four weeks (Table III and Figure 2). There were no statistically significant changes over time in the interaction analyses between groups dividing into strata of more or less than four hours crying at baseline (p = 0.20), the eventual influence of other types of treatment (p = 0.30), type of feeding (p = 0.97), or concerning the primary blinding validation question (p = 0.98). Corrected for baseline differences, there was a small tendency in favour of the acupuncture group, with a non-significant baseline-corrected sum of mean differences in crying time during the assessment period of 13 minutes (95% CI –24 to + 51). The effect size for the intervention in this trial was estimated to be 0.16.

**Table II.** Baseline-corrected mean overall difference in crying time reduction*: The ST36 infantile colic acupuncture trial.

	Total mean difference (minutes)	Sig.	95% Confidence Interval	
			Lower	Upper
Acupuncture Group/ Control group	13.25	0.482	–23.99	50.50

*Linear mixed model - Estimates of fixed effects.

**Table III.** Minutes of crying per day and differences between the acupuncture and control group (mean and 95% CI) at measured intervals: The ST36 infantile colic acupuncture trial.

	Acupuncture group (n = 38)	Control group (n = 41)	Difference (Cl)	p-value
Baseline (mean of day 1 and 2)	220 (195,245)	212 (183,241)	8 (–29,46)	0.67
1st intervention day (day 3)	230 (180,281)	186 (142,230)	44 (–21,110)	0.18
2nd intervention day (day 4)	180 (128,232)	185 (131,239)	–5 (–79,68)	0.88
3rd intervention day (day 5)	129 (97,162)	167 (123,211)	–38 (–91,17)	0.17
1 day after intervention (day 6)	120 (94,145)	142 (106,179)	–22 (–67,21)	0.31
1 week after interveniton (day 12)	121 (79,164)*	120 (91,149)*	1 (–49,52)	0.95
4 weeks after intervention (day 33)	89 (55,123)*	97 (67,126)‘	–8 (–51,37)	0.74

*One diary in each group did not give data for day 12 and day 33. For these days the acupuncture group n = 37, and the control group n = 40.

### Secondary outcomes

On day 6, nine out of 38 in the acupuncture group and 10 out of 41 in the control group fulfilled Wessel's criteria of more than three hours’ crying per day (odds ratio 1.1, CI 0.4–3.2, p = 0.94). This lack of differences was repeated at day 12 (p = 0.60) and day 33 (p = 0.31). The parents’ evaluation showed a slight tendency in favour of acupuncture over time, with a statistically significant difference on day 33 of 0.51 (CI 0.04–0.99), (p = 0.034). There were no serious adverse effects in the acupuncture or control group.

## Discussion

The trial showed a non-significant baseline-corrected mean difference of 13 minutes for the main outcome variable, changes in crying time, in favour of the acupuncture group. A linear mixed model of time versus group gave p = 0.26. This is not statistically significant or clinically relevant according to the protocol.

We failed to reach our sample size goal of 120 crying time diaries. With a baseline-corrected mean difference of 13 minutes in our trial and a standardized mean difference (SMD) or effect size of 0.16, we would have needed ten times as many participants, i.e. 1200 infants in a two-sample t-test for one time measure and 800 in a mixed model analysing changes over time, in order to show a statistically significant effect.

Needle-specific acupuncture effect for various pain conditions in adults have in meta-analyses turned out to be small, ranging from 0.15 to 0.23 [22,23]. Clinical acupuncture effects in adults are divided into three parts: cortical anticipation/reward (placebo) effects, non-specific physiological effects, and needle-specific effects.

Infants can be considered to have no anticipation/reward effects regarding acupuncture treatment. The effects the parents observe are the true needle effects, on the condition that the parents are truly blinded, as was the case in the present trial. The effect size of 13 minutes and SMD 0.16 is similar to the needle effect size found in adults, and contradicts the often implied notion by practitioners and textbooks that acupuncture effects in small children are stronger and faster [24–26].

There is only one review regarding acupuncture effects on pain conditions in infants, with only four eligible RCTs [27]. The authors conclude that the data are sparse, and that acupuncture on infants should be limited to clinical trials. There are no estimations of needle-specific effect sizes.

Two previous trials investigate acupuncture treatment in infantile colic, by Reinthal et al. and Landgren et al. [16,17]. Both trials concluded that acupuncture for infantile colic may reduce crying intensity and crying duration. These trials had different primary endpoints, and used different acupuncture points and different insertion time and method. The median differences in reduction in crying time in favour of the acupuncture group over the assessment period were 19 minutes (n = 40) and 11 minutes (n = 80). The corresponding result in our trial (n = 79) was a mean difference of 13 minutes, also in favour of the acupuncture group. So the estimated effect sizes in these three trials are similar, and small. Our choice of acupuncture points and technique could be inferior to other more potent acupuncture approaches. There are as yet no trial data on infants to support this. In adults, however, individual patient data analysis of acupuncture for various chronic pain conditions, with over 18 000 patients in total, showed effect sizes that were similar across groups and with different treatment approaches [23].

Acupuncture treatment in children is considered a safe intervention [15,28], which was confirmed in the present trial. Adverse reactions were few and insignificant.

Acupuncture in children is a potentially painful treatment [27], and this concern is especially important in small children without competence of consent [29]. Nevertheless, painful or potentially painful interventions in small children are used when the benefit clearly outweighs the harm. This consideration should also apply to acupuncture treatments. Acupuncture has been shown to work consistently in pain conditions in adults [23,30]. The needle-specific effects are small, and the combination of needle-physiological effects and cortical anticipation/reward effects contributes to improvement in several pain conditions. Small children have no established anticipation/reward systems; it is the real effect of the needle that defines the relevance of acupuncture treatment. It would be unethical to treat in response to the parents’ hope of improvement if the effect size does not outweigh the potential pain inflicted on the child. Our trial could not prove such a justification.

## Conclusion

Our trial of acupuncture for infantile colic showed no statistically significant difference in reduction of crying time between the acupuncture and the control group. There was a non-significant baseline-corrected mean overall difference in crying time reduction of 13 minutes in favour of the acupuncture group, which is not considered clinically relevant. With the current evidence, we suggest that acupuncture for infantile colic should be restricted to clinical trials.
